# Pyrocardan® interpositional arthroplasty for trapeziometacarpal osteoarthritis: a minimum four year follow-up

**DOI:** 10.1007/s00264-022-05457-3

**Published:** 2022-06-08

**Authors:** Francesco Smeraglia, Morena Anna Basso, Giulia Famiglietti, Andrea Cozzolino, Giovanni Balato, Alessio Bernasconi

**Affiliations:** Department of Public Health, Division of Orthopaedic Surgery, Federico II” University, Via S. Pansini 5, bd. 12, 80131 Naples, Italy

**Keywords:** Thumb, Arthritis, Pyrocarbon, Implant, Arthroplasty, Trapeziometacarpal joint

## Abstract

**Background:**

Pyrocardan® (Wright Medical-Tornier) is a pyrocarbon implant proposed in the treatment of trapeziometacarpal joint (TMCJ) osteoarthritis. Our aim was to assess the clinical and radiographic results after Pyrocardan® arthroplasty at midterm follow-up.

**Methods:**

In this prospective monocentric study, we enrolled 119 patients treated with Pyrocardan® for TMCJ osteoarthritis and followed up at a minimum of four years. The clinical outcome was assessed through the Disabilities of the Arm, Shoulder and Hand (DASH) questionnaire, the Visual Analog Score (VAS) for pain and the Kapandji score collected pre-operatively, at three, six and 12 months, then yearly. Hand radiographs were taken before surgery, at three months and every year. Complications and re-operations were also recorded.

**Results:**

The mean follow-up was 5.2 years (range, 4–9). DASH, VAS and Kapandji scores significantly improved at three (*p* < 0.001 in all cases) and six months (*p* < 0.001, *p* = 0.01 and *p* < 0.001, respectively), remaining stable over time. The dislocation and subluxation rates were 3.3% (4 cases) and 16.8% (20 patients), respectively. The two year, four year and seven year survivorship of the implant was 99%, 98% and 95%, respectively.

**Conclusion:**

Pyrocardan® arthroplasty provides a satisfactory clinical and radiographic outcome for treating TMCJ osteoarthritis, with a 97% survival rate at four years. We advocate comparative studies with more common techniques (i.e., trapeziectomy) to verify its cost-effectiveness.

## Introduction

Osteoarthritis of the trapeziometacarpal joint (TMCJ) may cause pain, weakness of the pinch, deformity and disability. When the conservative treatment fails, several surgical techniques have been proposed, but none has been proven superior to another [1–3]. Among them, trapeziectomy and its modifications are the most widely used, even if the proximal migration of the first metacarpal and the consequent loss of strength may represent an important drawback of the technique, particularly in young and highly demanding populations [1]. On the other side, joint replacement is an attractive option as a way to preserve the length of the first ray, resulting in a stronger pinch with a complete range of motion. Multiple implants have been described in literature, but to the best of our knowledge, no gold standard has been defined yet [4–6].

In 2011, Bellemère et al. introduced the Pyrocardan® (Wright Medical-Tornier) as a pyrocarbon implant which behaves as an intra-articular interposition [7] in the treatment of TMCJ osteoarthritis. While its biconcave surfaces would convert the saddle joint of the TMCJ into a cardan one, its structure in pyrocarbon would be theoretically advantageous given the elastic module similar to cortical bone [6, 8–10]. Encouraging results have been reported at over two years from surgery [11, 12], and only one study has documented a 96% survival rate at five years from surgery [13].

In this scenario, we set out to prospectively report the clinical and radiographic results of Pyrocardan® implant in the treatment of TMCJ osteoarthritis at a minimum follow-up of four years in order to confirm or disprove previous findings. We also focused on the complication and re-operation rates. Our hypothesis was that the clinical improvement in patients treated with Pyrocardan® would remain stable over time.

## Methods

### Study design

A prospective study was conducted for patients who received a Pyrocardan® implant between 2012 and 2017 at our institution. Surgery was performed by two experienced hand surgeons (levels 5 and 4, respectively [14]). Local ethical committee approval was not required for this observational study. No external funding was received for this study. Informed consent was obtained from all patients included in the study. Procedures were performed in accordance with the Helsinki declaration as revised in 2013.

### Enrolling criteria

Inclusion criteria were as follows: patients aged 15–85 years, failure of non-operative treatment after at least six months, pain at the TMC joint and radiological staging II or III (according to Eaton-Littler [15]). Involvement of the scaphotrapezium-trapezoid (STT) joint was an exclusion criterion. In the timeframe selected, Pyrocardan® was used in 137 patients. Out of them, 119 (86%) were available at a minimum four year follow-up. Patient demographics are reported in Table 1.

**Table 1:** Patients demographic and characteristics

Patients	119
Mean age (years)	60 (range 37–80 y)
Female (number, %)	96 (80.6%)
Right-handed (number, %)	61 (51%)
Mean follow-up (months)	60.9 (range 40–84 m)
Eaton-Littler classification (number, %)	
Stage II	88 (74%)
Stage III	31 (26%)

### Surgical technique

A Brachial plexus block was performed, and a high-arm tourniquet was inflated to 250 mmHg. A dorsal curvilinear incision was made in the TMCJ. The dorsal capsule was approached through the abductor pollicis longus and the extensor pollicis brevis. A trapezium-based flap was raised, and the joint was exposed subperiosteally to leave the capsule intact for direct closure.

A sagittal saw was used to perform the minimal resection of the metacarpal base and trapezium. A meticulous resection of the osteophytes was performed, and the horns of the trapezium were resected. A spherical burr was used to reduce the irregularity and remodel the two surfaces. The trial implant was inserted and assessed using fluoroscopy. Finally, the size of the implant which fully covered the trapezium surface was chosen. The trial was replaced with a definitive implant. A suture of the capsule was performed with a transosseous suture on the first metacarpal base. Postoperatively, a thumb spica cast was applied. At two weeks post-operatively, the sutures were removed, and a removable splint was provided for gradual use of the hand. Rehabilitation was started at week three, and unrestricted activities were allowed after week six.

### Clinical assessment

Patients were assessed pre-operatively (T0) and post-operatively at three months (T1, *N* = 119), six months (T2, *N* = 119), one year (T3, *N* = 119), two years (T4, *N* = 118), three years (T5, *N* = 118), four years (T6, *N* = 117), five years (T7, *N* = 76), six years (T8, *N* = 36) and seven years (T9, *N* = 25). Patients completed the Disabilities of the Arm, Shoulder and Hand (DASH) questionnaire score (0 points no disability; 100 points complete disability) to assess the function of the upper limb [16]. Pain was assessed using a 10-cm VAS. The scale was graded from 0 to 10 cm, with 0 cm indicating no pain and 10 indicated maximum pain. Thumb motion was assessed using the Kapandji test [17], with 1 indicating incapacity of opposition and 10 indicating complete opposition and measuring radial and palmar abduction with the help of a goniometer. Key-pinch strength was measured with a Jamar pinch dynamometer (FEI, Irvington, NY, USA) on both the operated and non-operated hands. Clinical assessments were performed by a single orthopaedic resident with adequate training in hand surgery. Complications and re-operations were also recorded.

### Radiographic assessment

**Fig. 1:**
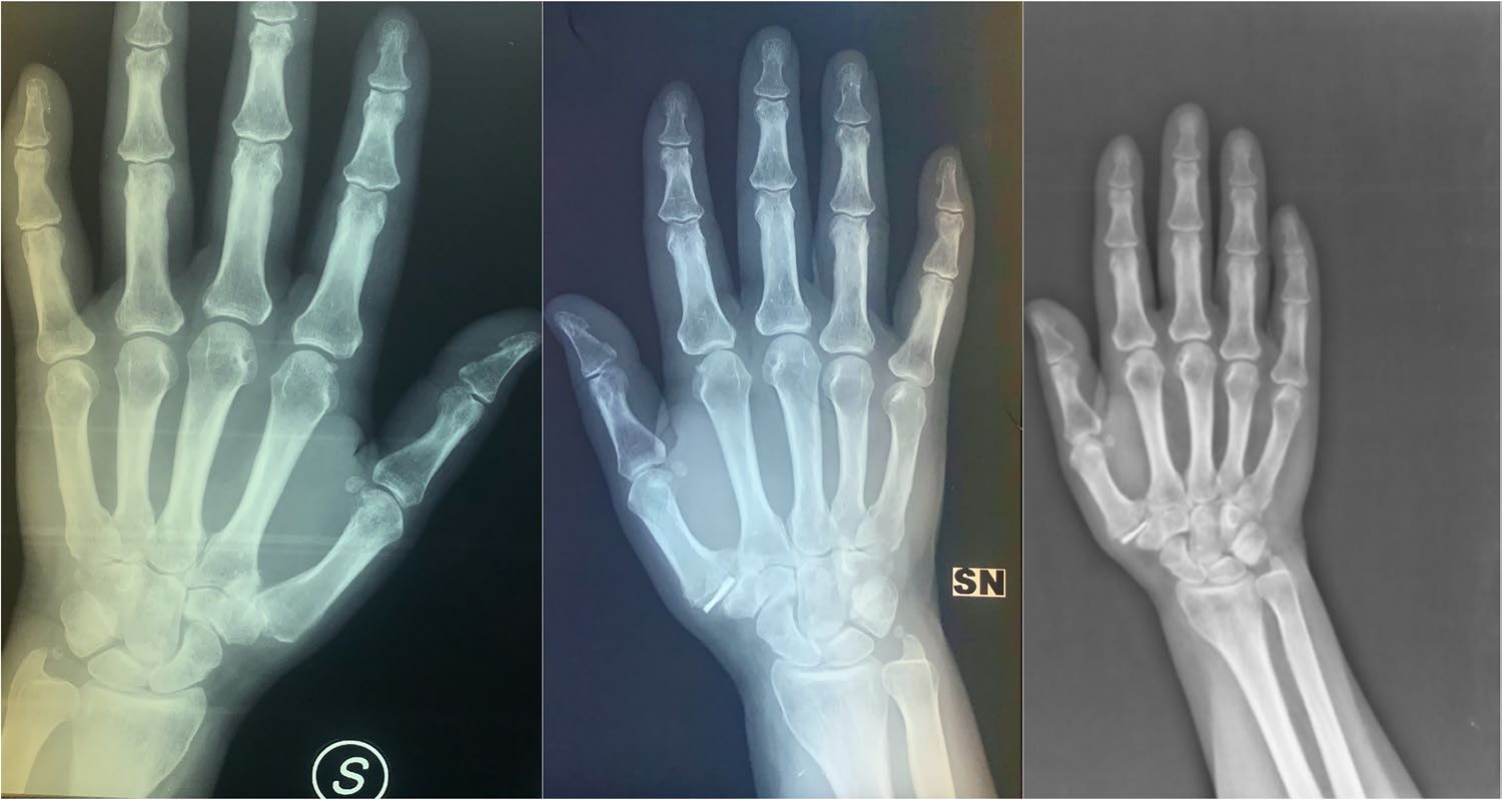
Woman, 64 years old, pre-operative X-ray, post-operative X ray at 3 months and post-operative X-ray at last follow-up of 5 years

Anteroposterior (AP) and lateral radiographic views of the TMCJ were obtained pre-operatively and during follow-up at three months and every year (Fig. 1), and the Eaton–Littler radiographic classification system [15] was used to stage thumb TMCJ osteoarthritis pre-operatively. Subluxation and dislocation were defined as a partial (more than one-fourth of the metacarpal base displaced) or complete loss of positioning of the implant, respectively, as previously reported [9].

### Statistical analysis

Normality of data was assessed using the Shapiro–Wilk test. Multi-comparison tests were performed with analysis of variance (ANOVA) test for repeated measures into groups, and the Bonferroni correction (*B*) of *p*-value was used in pairwise comparison into groups to assess differences in DASH, VAS, Kapandji score, ABD-P, ABD-R and key-pinch at different control points (T0, T1, T2, T3, T4, T5 and T6). Univariate analyses were performed to assess the association of age (Pearson’s correlation), sex and side (Wilcoxon rank sum analysis) and the position of the implant at the longest follow-up as normal, subluxed or dislocated (Kruskal–Wallis test) against the following continuous variables: (1) DASH, VAS Kapandji score and key-pinch at different times (T0 and T6); (2) the improvement in DASH, VAS, Kapandji score and key-pinch at T6 (delta = T6–T0). Variables found to be independently significant in the univariate analyses were then included in a multivariable linear regression analysis to determine the predictors of clinical outcome. Kaplan–Meier survival curves were drawn, with the event of interest being any revision surgery, at one, two and four years of follow-up. The analysis was performed using the STATA statistical software package version 12.0 (StataCorp, College Station, TX, 2011). The *p*-value was set to 0.05.

## Results

### Clinical outcome

The mean follow-up in the study was 5.2 years (range, 4–9 years). DASH, VAS and Kapandji scores significantly improved at three (*p* < 0.001 in all cases) and six months (*p* < 0.001, *p* = 0.01 and *p* < 0.001, respectively), remaining stable at the longest follow-up. While palmar abduction improved at  six months (*p* = 0.002), radial abduction and key-pinch showed a slower recovery trend that was significantly different from the immediate post-operative values only after 1 year (*p* = 0.006 and *p* = 0.005, respectively) (Table 2). Age and side did not significantly correlate with pre-operative values, last follow-up values and the improvement achieved in different scores. Conversely, males demonstrated a greater key-pinch at baseline (*p* < 0.001) and at the last follow-up (*p* < 0.001), with a more pronounced improvement after surgery (*p* = 0.02).

**Table 2:** Clinical parameter scores (mean) for all the patients at T0 (pre-operative), T1 (3 months), T2 (6 months), T3 (1 years), T4 (2 years), T5 (3 years), T6 (4 years), T7 (5 years), T8 (6 years) and T9 (7 years). Multi-comparison tests were performed with ANOVA test for repeated measures into groups, and the Bonferroni correction (*B*) of *p*-values was used in pairwise comparison into groups between two consecutive control points

	T0 (*N* = 119)	T1 (*N* = 119)	T2 (*N* = 119)	T3 (*N* = 119)	T4 (*N* = 118)	T5 (*N* = 118)	T6 (*N* = 117)	T7 (*N* = 76)	T8 (*N* = 36)	T9 (*N* = 25)
Parameter										
DASH score	59.6	37.6	25.3	19.8	17.8	16.6	15.9	16.2	21.7	16.3
*p-value*		T0 vs T1: < 0.001 (*B*)	T1 vs T2: < 0.001 (*B*)	T2 vs T3: 1 (*B*)	T3 vs T4: 1 (*B*)	T4 vs T5: 1 (*B*)	T5 vs T6: 1 (*B*)	T6 vs T7: 1 (*B*)	T7 vs T8: 1 (*B*)	T8 vs T9: 1 (*B*)
VAS score	8.4	4.1	2.9	2.3	2.2	2.2	2.2	2.4	3.1	2.4
*p-value*		T0 vs T1: < 0.001 (*B*)	T1 vs T2: 0.01 (*B*)	T2 vs T3: 1 (*B*)	T3 vs T4: 1 (*B*)	T4 vs T5: 1 (*B*)	T5 vs T6: 1 (*B*)	T6 vs T7: 1 (*B*)	T7 vs T8: 1 (*B*)	T8 vs T9: 1 (*B*)
Kapandji test	8.7	7.8	8.9	9.1	9.1	9.2	9.2	9.2	9.2	9.2
*p-value*		T0 vs T1: < 0.001 (*B*)	T1 vs T2: < 0.001 (*B*)	T2 vs T3: 1 (*B*)	T3 vs T4: 1 (*B*)	T4 vs T5: 1 (*B*)	T5 vs T6: 1 (*B*)	T6 vs T7: 1 (*B*)	T7 vs T8: 1 (*B*)	T8 vs T9: 1 (*B*)
Palmar abduction	57	54.5	59.2	60.8	61.3	61.3	61.5	63.2	64.3	64.7
*p-value*		T0 vs T1: 1 (*B*)	T1 vs T2: 0.002 (*B*)	T2 vs T3: 1 (*B*)	T3 vs T4: 1 (*B*)	T4 vs T5: 1 (*B*)	T5 vs T6: 1 (*B*)	T6 vs T7: 1 (*B*)	T7 vs T8: 1 (*B*)	T8 vs T9: 1 (*B*)
Radial abduction	57.7	56	59	60.4	60.8	60.8	61.2	62.6	63.7	64.8
*p-value*		T0 vs T1: 1 (*B*)	T1 vs T2: 0.386 (*B*)	T2 vs T3: 1 (*B*)	T3 vs T4: 1 (*B*)	T4 vs T5: 1 (*B*)	T5 vs T6: 1 (*B*)	T6 vs T7: 1 (*B*)	T7 vs T8: 1 (*B*)	T8 vs T9: 1 (*B*)
Key-pinch	2.8	2.9	3.4	3.5	3.6	3.6	3.6	3.6	3.7	4.3
*p-value*		T0 vs T1: 0.001 (*B*)	T1 vs T2: < 0.001 (*B*)	T2 vs T3: 1 (*B*)	T3 vs T4: 1 (*B*)	T4 vs T5: 1 (*B*)	T5 vs T6: 1 (*B*)	T6 vs T7: 1 (*B*)	T7 vs T8: 1 (*B*)	T8 vs T9: 1 (*B*)

### Radiographic outcome

**Fig. 2:**
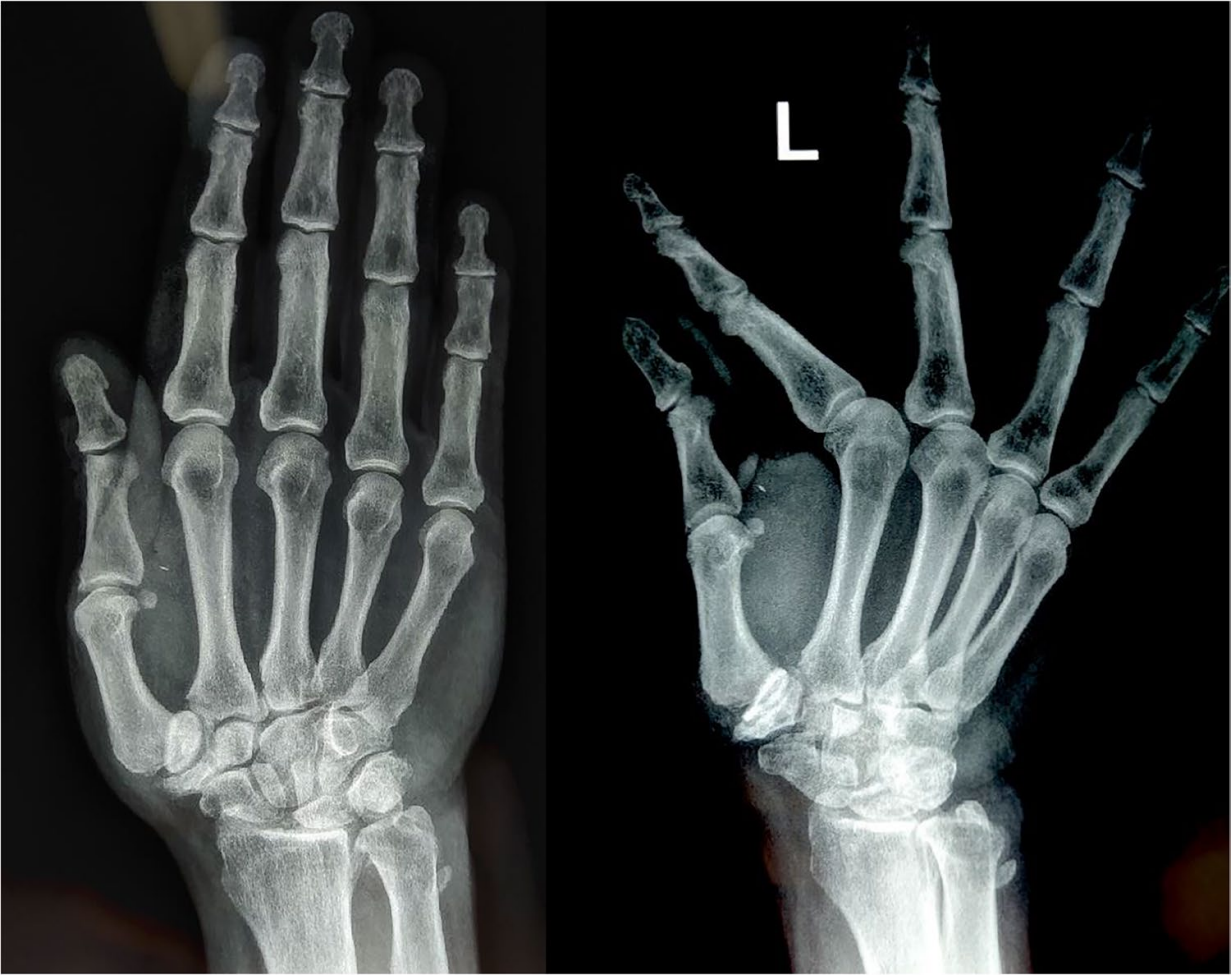
Man, 72 years old, post-operative X-ray at 4-year follow-up with a subluxation of more than 1/4 of the metacarpal base

Dislocation and subluxation of the implant were found in four (3.3%) and 20 patients (16.8%), respectively (Fig. 2). Subluxed or dislocated implants were found to be associated with pain (VAS) and limited ROM as radial and palmar abduction and with a reduced improvement after surgery. In these cases, the change according to the Kapandji score was significantly lower than that in normally positioned implants (*p* = 0.03). Among these 24 patients, only three of them (all dislocated implants) required revision surgery and were treated with implant removal and trapeziectomy. After a follow-up of three, seven and eight years (for the 3 patients) from the revision procedure to trapeziectomy, DASH (17, 15 and 23 points, respectively) and VAS (1, 0 and 2, respectively) were satisfactory.

### Complication and re-operation rate

**Fig. 3:**
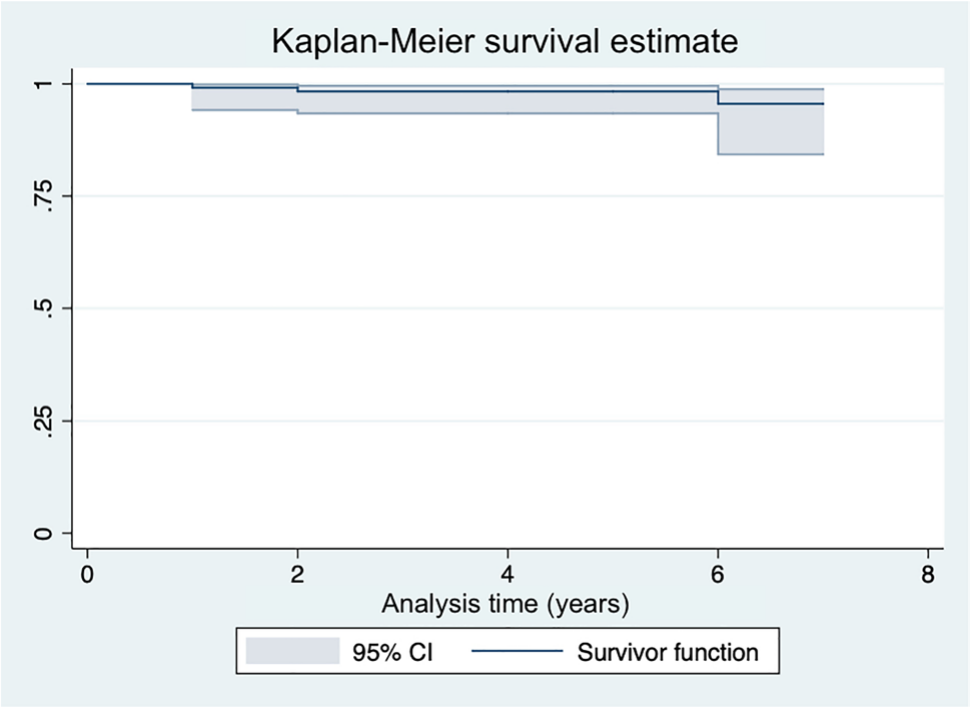
The Kaplan–Meier curve showing the implant survival rate

None of the patients reported any intra-operative complications. Nine patients (7.5% of the population) experienced apraxia of the radial sensitive nerve which resolved within three months without treatment. The Kaplan–Meier curve showed an implant survival rate of 99% at one year, 98% at two and five years and 95% at seven years (Fig. 3; Table 3).

**Table 3:** Data on Pyrocardan® survival rate

Time (years)	Patients	Survivor function	[95% Conf. Int.]
1	119	0.99	0.94–0.99
2	118	0.98	0.93–0.99
3	118	0.98	0.93–0.99
4	117	0.98	0.93–0.99
5	76	0.98	0.93–0.99
6	36	0.95	0.84–0.98
7	25	0.95	0.84–0.98

## Discussion

The main finding of this study was that patients treated with Pyrocardan® for end-stage TMCJ osteoarthritis experienced a significant and long-lasting clinical improvement after surgery, with an overall low complication and re-operation rate. In the majority of patients, a satisfactory outcome remained stable over time, with a survivorship of Pyrocardan® standing at 95% at seven year follow-up. The instability of the implant, with subluxation or dislocation, was obviously associated with a poorer outcome, but in this series could be successfully tackled through a revision to trapeziectomy.

A few previous studies have investigated the efficacy of Pyrocardan® to treat TMCJ osteoarthritis [7, 11–13, 18–20] (Table 4). In these studies, authors have already outlined the advantages related to Pyrocardan®, i.e. the minimal resection of articular surfaces which prevents an excessive shortening of the first ray, the preservation of the range of motion (as compared to arthrodesis) and the possibility to perform a revision to trapeziectomy in case of failure of the implant [7, 11–13, 18–20]. To date, the study with the longest follow-up has been published by Gerace et al., who documented a significant improvement after pyrocarbon interposition arthroplasty in the pain level, QuickDASH and strength, with a 4% revision rate and a 96% survival rate at 5 years follow-up [13]. While the clinical results in our series confirm these findings, we believe that at least two important differences between this study and the one by Gerace et al. have to be outlined. First, the prospective design in our analysis allowed to overcome the biases inherently related to retrospective studies. Second, we were able to assess patients on a year-by-year basis, estimating the survivorship of the implant over time and reporting a 95% survival rate seven years after surgery, which to the best of our knowledge is the longest follow-up reported in literature. We believe that clinicians should explain these data to patients during the pre-operative counselling, in order to allow a correctly informed decision about the procedure.

**Table 4:** Outcomes of Pyrocardan® for the treatment of TMCJ osteoarthritis in previous published studies

Authors	Year	Implant	*n*	Mean age (years)	Mean follow-up (years)	Quick-DASH or DASH	VAS	Tip or key-pinch (kg)	Grip strength (kg)	Satisfaction rate	Survival rate	Complication rate	Revision rate	Dislocation rate	Subluxation rate
Our study	2021	Pyrocardan	119	60	5	16.3	2.4	4.3	NR	NR	97%	7.5%	2.5%	3.3%	16.8%
Gerace et al	2020	Pyrocardan	103	59	5.5	9	0.6	7	27	96%	96.2%	4.8%	3.8%	0	0
Logan et al	2020	Pyrocardan	40	58	2.5	23	1.7	5	30	83%	100%	NA	0%	0	0
Erne et al	2017	Pyrocardan	8	64.3	1.5	18.3	1.5	0.73 bar	NA	7.4/10	100%	NA	12%	0	0
Lauwers et al	2016	Pyrocardan	28	59	2	NR	NA	NR	NR	75%	NA	NR	18%	10.7%	0
Russo et al	2016	Pyrocardan	40	58.5	2.5	18.7	2.7	4.6	NR	NR	94.5%	NA	5%	5%	0
Odella et al	2014	Pyrocardan	25	55	1	22.4	4	NR	NR	NR	88%	12%	8%	0	0
Belleme` re et al	2011	Pyrocardan	27	58	1.5	10.1	1.3	6.7	NA	NR	100%	NA	0%	0	0

With regard to re-operations, in this study, revision was necessary in 3% of cases, which is in keeping with values reported in previous literature (ranging from 0 to 18%) [7, 11–13, 18–20]. All patients needing revision presented with a dislocation of the implant, which in our opinion may be related to the instability inherently related to Pyrocardan®. In a series by Herren et al. [21], authors reported a less favourable outcome after revision surgery as compared to primary trapeziectomy. Conversely, it should be emphasized that in our series, patients who undergone trapeziectomy reported clinical results overlapping those reported after primary trapeziectomy (18.3 points for DASH as compared to 17–34 points reported in a recent systematic review about trapeziectomy [22]). Studies reporting data on larger cohorts of ‘revised’ patients are needed to confirm or disprove these findings.

For what concerns other treatment available in the treatment of TMCJ osteoarthritis, trapeziectomy remains the gold standard treatment. Since the clinical results reported in literature are similar to ours in terms of pain relief and function [23–29], a question arises about the cost-effectiveness of Pyrocardan® as compared to trapeziectomy which is intuitively a cheaper procedure. We advocate dedicated prospective comparative cost-analyses between these two treatments in order to shed light on the superiority of a technique over one other. On a different note, trapeziometacarpal arthrodesis still has a place in the armamentarium of hand surgeons, but it is not recommended as first-line option due to the significant loss of motion and the onset or progression of scapho-trapezial joint osteoarthritis [30, 31].

The authors acknowledge some limitations of this study. First, lack of a group of control. Second, 14% of patients (18/137) did not reach the minimum four year follow-up originally set in the study protocol and were lost at follow-up; although a formal power analysis was not carried out, we reckon that a final sample size of over 100 patients with a minimum follow-up of four years might be sufficiently informative to report midterm results of Pyrocardan®. Third, as discussed above, we did not perform a cost-effectiveness analysis, which would have provided paramount data in order to draw conclusions on the best surgical treatment for TMCJ osteoarthritis.

## Conclusion

In conclusion, the use of Pyrocardan® in TMCJ osteoarthritis provides pain relief associated with a satisfactory functional outcome which persist over time. The estimated survival rate at 7 years from surgery stands at 95%. In case of failure, revision surgery with conversion to trapeziectomy can be performed with good results. We advocate further comparative studies in order to shed some light on the cost-effectiveness of the implant as compared to other common procedures such as trapeziectomy with or without ligament reconstruction.

## Data Availability

Yes.
